# A pilot study: LASEK with the Triple-A profile of a MEL 90 for mild and moderate myopia

**DOI:** 10.1186/s12886-017-0493-4

**Published:** 2017-06-23

**Authors:** Yingjun Chen, Dong Yang, Tian Han, Haipeng Xu, Meiyan Li, Xingtao Zhou

**Affiliations:** 1 Department of Ophthalmology, Eye and ENT Hospital, Fudan University, Shanghai, China; 2 Carl Zeiss Meditec AG, Shanghai, China

**Keywords:** MEL 90, LASEK, Triple-A, Myopia

## Abstract

**Background:**

To investigate the visual and refractive outcomes in patients with mild to moderate myopia after laser-assisted subepithelial keratectomy (LASEK) using the 500 Hz pulse rate of the Triple-A profile.

**Methods:**

Thirty-six eyes of 20 patients (mean age, 27.5 ± 4.6 years) were included in this prospective, consecutive study. Uncorrected distance visual acuity (UDVA), corrected distance visual acuity (CDVA), corneal topography, and corneal aberrations were measured preoperatively and 1 day, 1 week, 1, 3 and 6 months post-operation.

**Results:**

At 1 week after surgery, UDVA was better than or equal to 20/25 in all eyes. At postoperative 6 months, the efficacy and safety index was 1.05 ± 0.13 and 1.12 ± 0.15, respectively; all eyes had a UDVA of 20/20 or better, and no eyes showed a loss in CDVA; 100% of the eyes were within ±1.00 D of the attempted spherical equivalent (SE) correction.

**Conclusion:**

The postoperative results indicate that using the Triple-A ablation profile of the MEL 90 excimer laser with a 500 Hz pulse rate is a safe, efficient, and predictable method to correct mild to moderate myopia.

## Background

The excimer laser has the potential of producing tissue ablation with a high degree of precision and with minimal damage to the adjacent structures [1]. Laser-assisted subepithelial keratectomy (LASEK) was introduced in 1999 by Camellin for the correction of refractive error [2]. Treatment for myopia and myopic astigmatism using the MEL 80 excimer laser (Carl Zeiss Meditec AG, Jena, Germany) with the tissue saving ablation (TSA) and aberration smart ablation (ASA) profiles during LASEK has been reported to have excellent refractive outcomes in safety, efficacy, and predictability [3–6]. The use of ASA can induce a positive spherical aberration when correcting for high myopia and a relatively higher ablation depth when treating low myopia [7]. The advanced ablation algorithm (Triple-A) profile, developed by Carl Zeiss Meditec AG, is an ideal ablation profile to achieve better visual outcomes with a lower induction rate of higher-order aberrations (HOAs) while decreasing the ablation depth for the correction of myopia, especially for low myopia [7–9]. The MEL 90 excimer laser has two frequencies available for the correction of refractive error. The 250 Hz pulse rate has been recommended to treat refractive error for surface ablation, while the 500 Hz pulse rate has been used in laser-assisted in-situ keratomileusis (LASIK) procedures. Reinstein et al. demonstrated that the 500 Hz pulse rate achieved high efficacy for myopia up to −10.00 D and cylinder up to 5.00 D after LASIK [9]. For LASEK procedures, the refractive outcomes with the MEL 90 excimer laser have not yet been reported. Therefore, we conducted a prospective study to demonstrate the efficacy and safety after LASEK using a 500 Hz pulse rate.

## Methods

### Subjects

The research was conducted at the Ophthalmology Department of the Eye and ENT Hospital in Shanghai, China. In this prospective study, 36 eyes of 20 consecutive patients who received LASEK using the MEL 90 excimer laser (the MEL 90 group) for the correction of myopia and myopic astigmatism were enrolled. The follow-up time was 6 months.

For the control group (the MEL 80 group), we included 33 eyes of 17 patients who were treated with the TSA profile using the 250 Hz pulse rate of the MEL 80 with 6.5 mm for the optical zone (Carl Zeiss Meditec, Jena, Germany). Data were selected from our previously published data.

Inclusion criteria were a spherical refraction less than −6.00D, astigmatism from 0.00 to −3.75D, corrected distance visual acuity (CDVA) of 20/25 or better, stable refraction for 2 years before surgery, and an absence of other pathologic ocular conditions or relevant systemic diseases.

The research followed the tenets of the Declaration of Helsinki and was approved by the Ethics Committee of the EENT Hospital of Fudan University. All patients signed a consent inform.

### Surgical technique

**Fig. 1:**
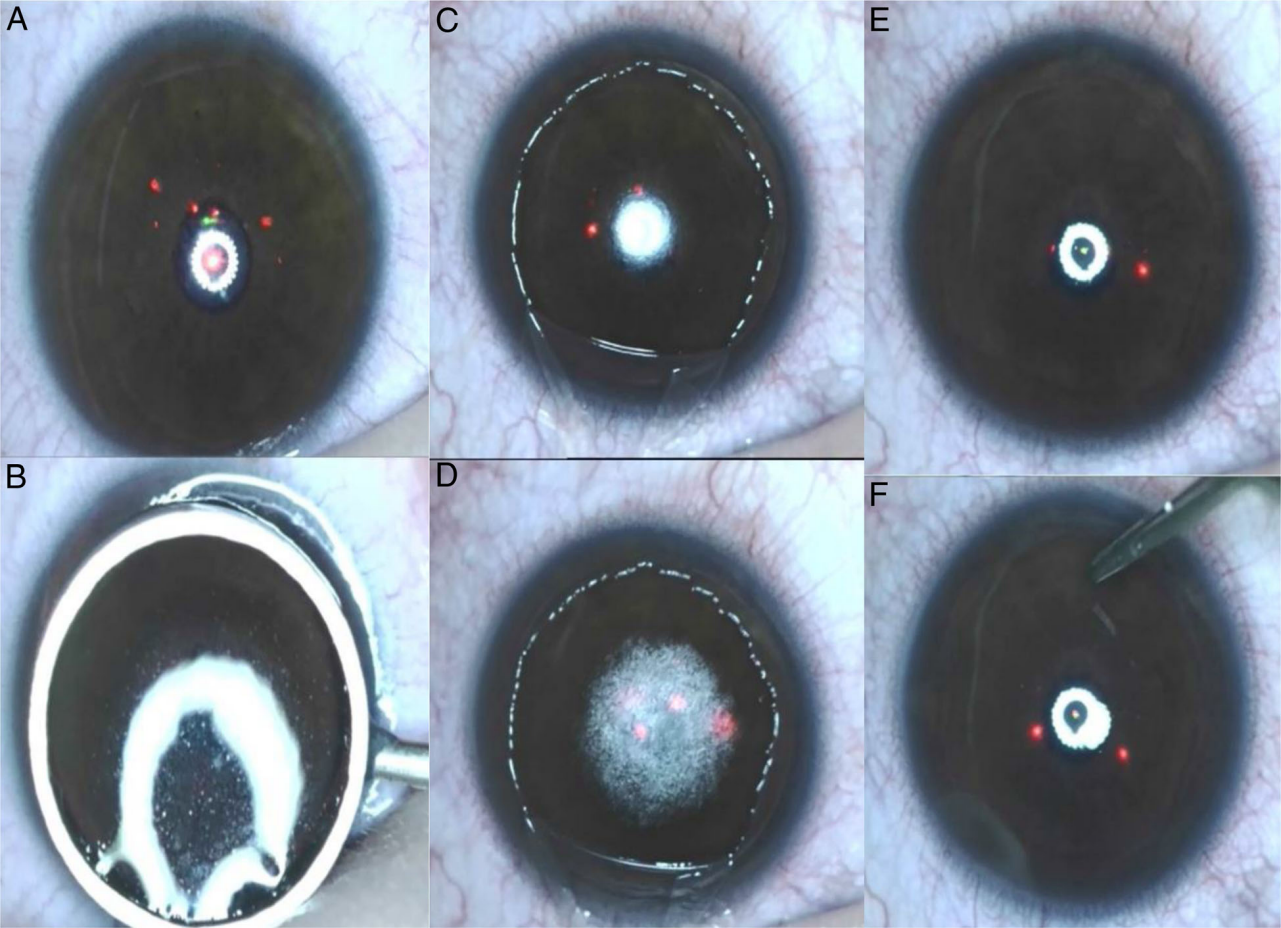
The represented images showed the laser-assisted subepithelial keratectomy (LASEK) surgical procedures. The ablation center adjustment (**a**); the corneal epithelium was immersed in 20% alcohol (**b**); the epithelial flap was removed following immersion (**c**); the Corneal Bowman’s membrane and stroma were ablated with the MEL 90 excimer laser (**d**); the epithelial flap was repositioned after laser ablation (**e**); a bandage soft contact lens was applied (**f**)

LASEK treatments began with 12 s of 20% alcohol-assisted epithelial flap creation, followed by standard excimer laser ablation using the MEL 90 with a 500 Hz pulse rate (Carl Zeiss Meditec, Jena, Germany, Triple-A profile, LASIK mode). The optical zone was 6.5 mm in all patients (Fig. 1). The epithelial flap was repositioned after laser ablation, and a bandage soft contact lens (ACUVUE OASYS; Johnson & Johnson, New Brunswick, NJ) was applied. The target refraction was plano for all patients.

### Postoperative evaluation

Patients were instructed to wear the bandage soft contact lens for 1 week. Topical steroids (fluorometholone 0.1%; Santen Pharmaceutical Co., Ltd.) were initially administered 8 times/day and tapered over 75 days. Topical antibiotics (ofloxacin ophthalmic solution 0.5%; Santen Pharmaceutical Co., Ltd.) were administered 4 times/day for 7 days. Tears Naturale (hypromellose 2910, dextran 70, glycerol eye drops; Alcon Laboratories, Inc.) were administered 4 times/day for 3 months.

Patients were followed-up at postoperative 1 day, 1 week, 1, 3 and 6 months. All subsequent follow-up visits recorded uncorrected distance visual acuity (UDVA), CDVA, manifest refraction, corneal topography, and corneal aberrations. All postoperative examinations were conducted by the same experienced optometrists.

### Measurement of corneal aberrations

Corneal HOAs were obtained using a rotating Scheimpflug Camera (Pentacam HR; Oculus, Wetzlar, Germany) in a dark room. The corneal HOAs of the anterior surface, posterior surface, and total cornea were analyzed over a 6.0 mm central diameter at pre-operation and at postoperative 6 months. We analyzed the root mean square values of the corneal HOAs including the third to sixth HOAs.

### Data analysis

Statistical analyses were performed using SPSS software (version 13.0; SPSS Inc.). The mean ± standard deviation (SD) was calculated. A Student’s t-test was performed to determine the difference between pre-operation and post-operation and between the MEL 90 and MEL 80 groups. *P* values of less than 0.05 were considered statistically significant.

## Results

Table 1 shows the demographic data of the two groups. All surgeries were uneventful without any intraoperative complications. No postoperative complications, such as corneal haze, wound dehiscence, inflammation and infection were observed in any eyes.

**Table 1:** Study demographics

Parameter	MEL90 Group	MEL80 Group	*P*
No. of eyes	36 (20 patients)	33 (17 patients)	
Age, y (range)	27.5 (20 to 37)	30.6 (23 to 41)	
Gender ratio	40% F/60% M	47% F/53% M	
Attempted SE (range)	−3.50 ± 1.31 D (−1.13 to −5.63 D)	−3.45 ± 1.28 D (−0.75 to −5.63 D)	0.923
Attempted sphere (range)	−3.19 ± 1.38 D (−0.25 to −5.25 D)	−3.11 ± 1.40 D (−0.25 to −5.50 D)	0.793
Attempted cylinder (range)	−0.61 ± 0.46 D (0.00 to −1.75 D)	−0.73 ± 0.92 D (0.00 to −3.75 D)	0.503
CDVA	83% ≥ 20/16; 97% ≥ 20/20	39% ≥ 20/16; 97% ≥ 20/20	0.001
Follow-up	100% 6 months	100% 6 months	

*SE* spherical equivalent refraction, *CDVA* corrected distance visual acuity, *D* diopters

### Refractive outcomes

**Fig. 2:**
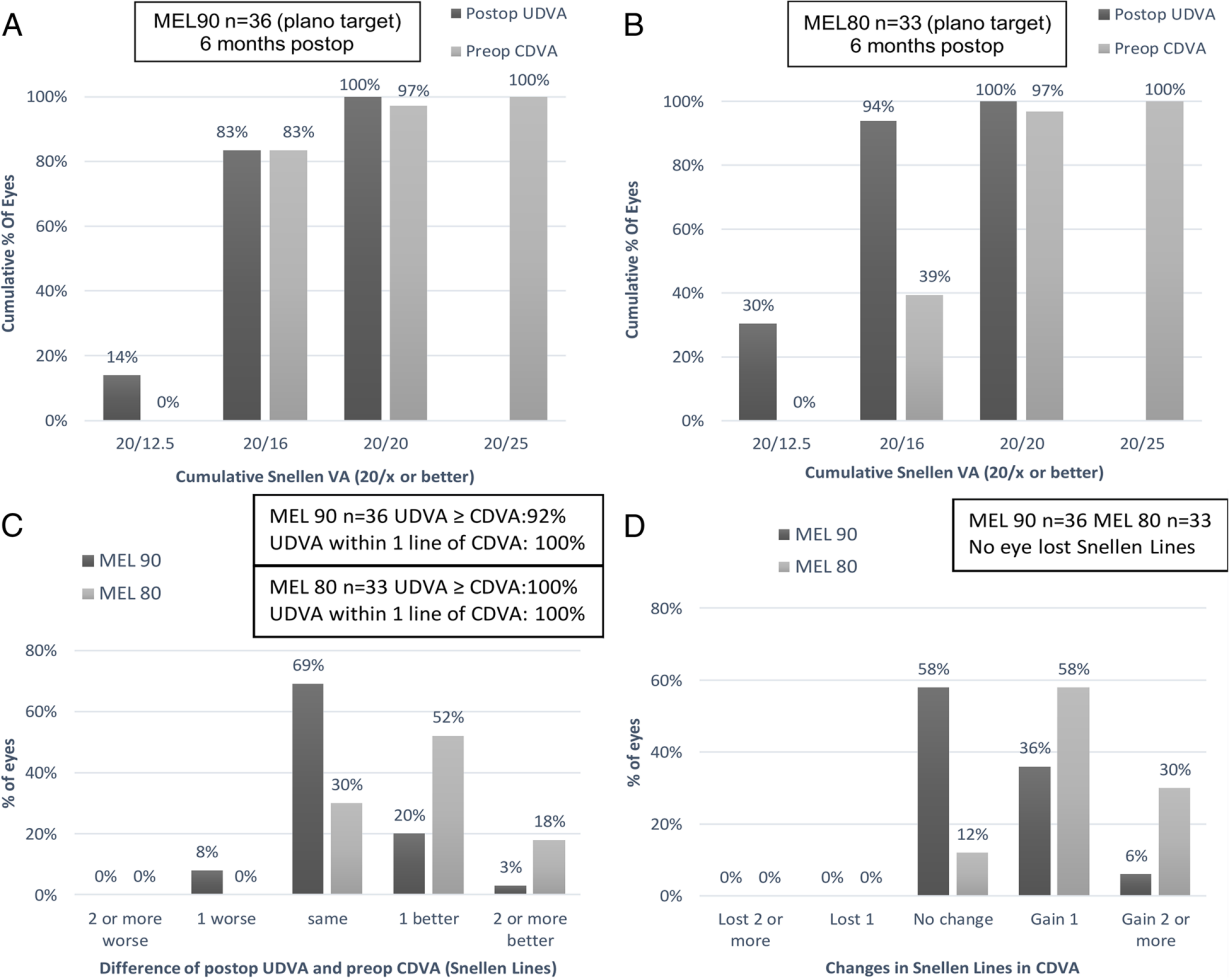
Visual outcomes after the MEL 90 and MEL 80 at 6 months: Cumulative percentage of eyes with preoperative corrected distance visual acuity (CDVA) and postoperative uncorrected distance visual acuity (UDVA) results (**a**&**b**), percentage of eyes with various changes in postoperative UDVA and preoperative CDVA (**c**), and percentage of eyes with various changes in CDVA (**d**)

At postoperative 6 months, the efficacy and safety index after MEL 90 was 1.05 ± 0.13 and 1.12 ± 0.15, respectively. Figure 2 shows the distribution of UDVA and CDVA following LASEK. Table 2 shows the manifest refraction, logMAR CDVA and logMAR UDVA after use of the MEL 90 and MEL 80 excimer laser at 6 months after surgery.

**Table 2:** Manifest refraction and log MAR UDVA and CDVA after LASEK in MEL90 and MEL80 groups

Parameter	MEL90 Group	MEL80 Group	*P*
MR sphere (D)	0.26 ± 0.36 D	0.29 ± 0.37 D	0.727
MR cylinder (D)	−0.22 ± 0.19 D	−0.19 ± 0.21 D	0.492
MRSE (D)	0.15 ± 0.41 D	0.19 ± 0.37 D	0.613
log MAR UDVA	−0.10 ± 0.06	−0.12 ± 0.06	0.052
log MAR CDVA	−0.13 ± 0.06	−0.16 ± 0.05	0.028

*MR* manifest refraction, *UDVA* uncorrected distance visual acuity

**Fig. 3:**
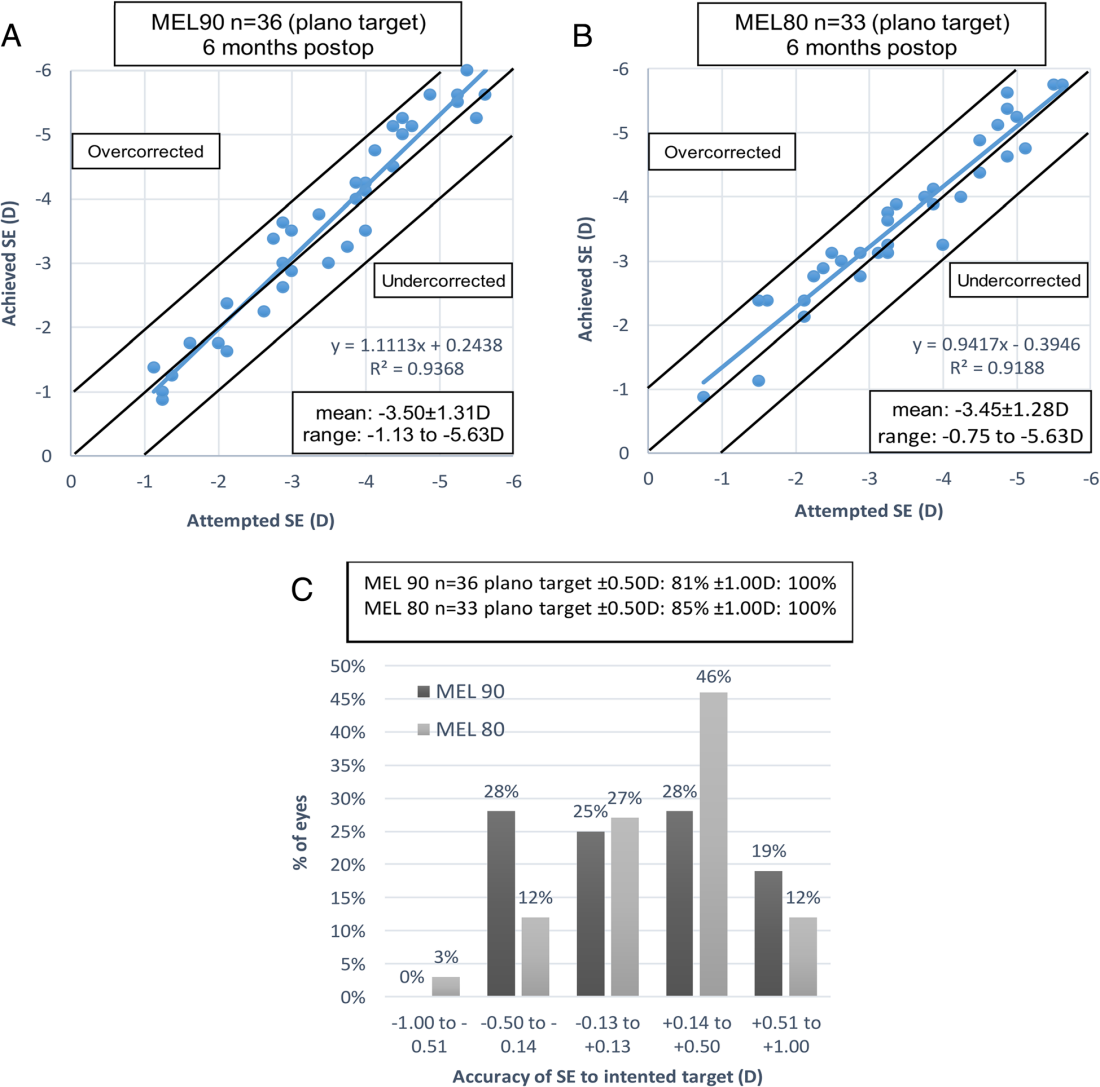
Scatterplot of the attempted versus achieved manifest spherical equivalent (SE) correction at 6 months after LASEK surgeries in both groups (**a**&**b**). Refractive outcomes at 6 months: Percentages of eyes within different diopter ranges of the intended correction in SE (**c**)

At postoperative 6 months, 81 and 85% of eyes were within ±0.50 D of the attempted spherical equivalent (SE) correction in the MEL 90 and MEL 80 groups, respectively, and 100% of eyes were within ±1.00 D of the attempted SE correction in both groups. Scatterplots of the achieved versus attempted spherical equivalent are shown in Fig. 3a and b . The percentage of eyes with the accuracy of SE to intended target is shown in Fig. 3c.

**Fig. 4:**
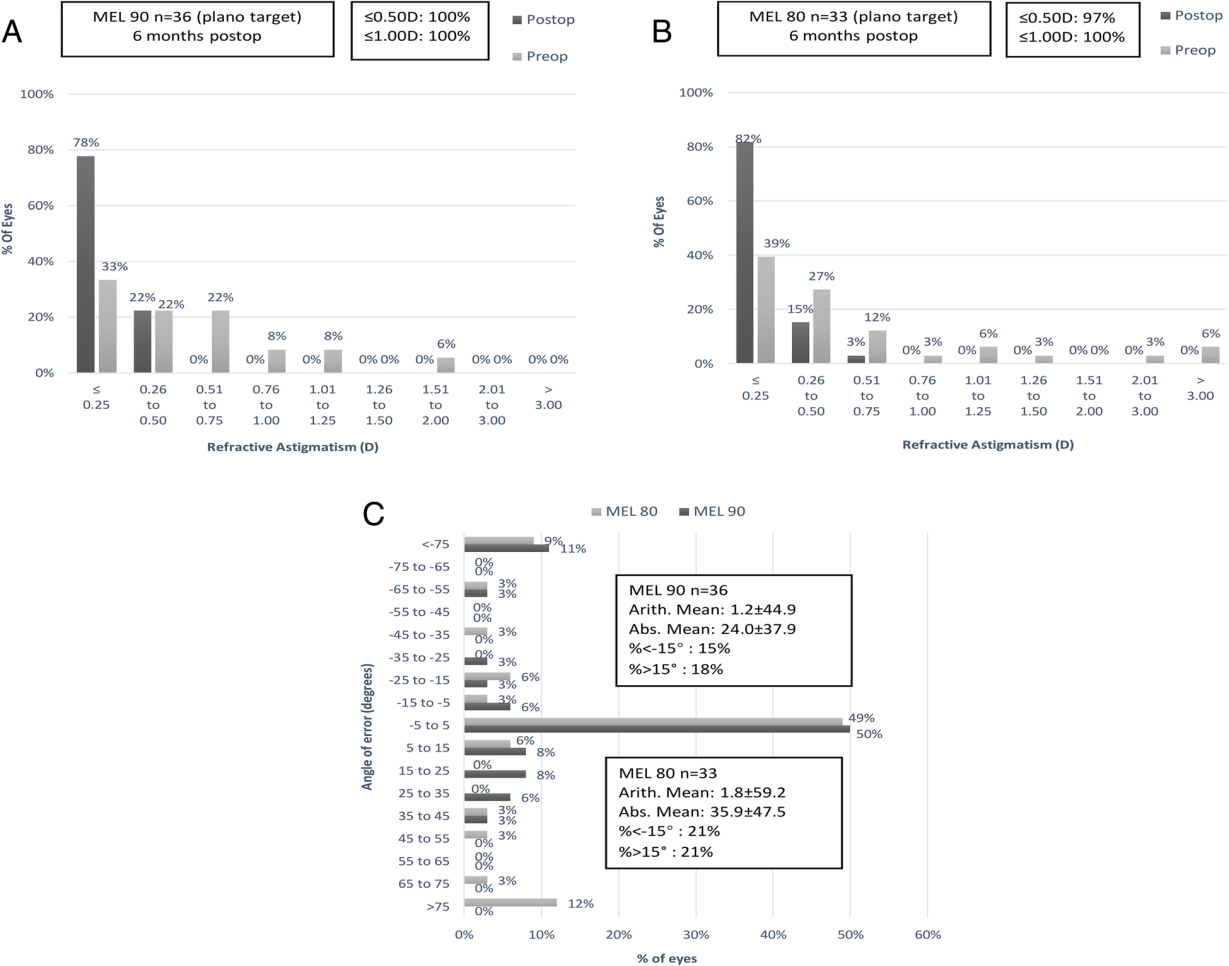
Vector analysis for LASEK at 6 months after surgery in both groups. Percentages of eyes that achieved definite levels of astigmatism (**a**&**b**), and distribution of the angle of error (**c**)

Figure 4a and b shows the percentage of eyes with refractive astigmatism. The distributions of absolute angle of error are presented in Fig. 4c.

### Corneal aberrations

**Fig. 5:**
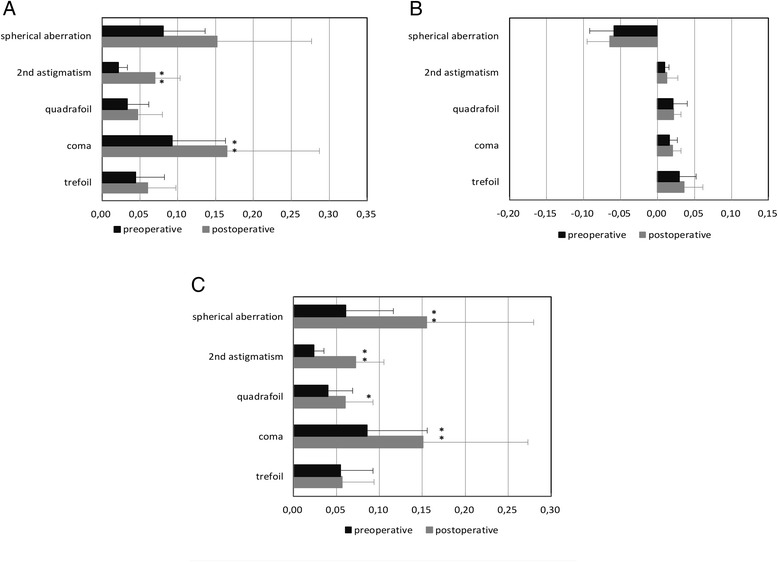
Changes in corneal aberrations pre-operation and at 6 months after surgery using the MEL 90 excimer laser. **a** Changes in anterior corneal aberrations (μm). **b** Changes in posterior corneal aberrations (μm). **c** Changes in total corneal aberrations (μm). ^*^ Less than 0.05, ^**^ less than 0.01

The mean change in anterior, posterior, and total corneal spherical aberration induction was 0.071 ± 0.112 μm, −0.005 ± 0.014 μm, and 0.094 ± 0.079 μm, respectively (Fig. 5).

## Discussion

Although LASIK has gained widespread acceptance in corneal refractive surgery due to little pain and rapid visual rehabilitation, surface ablation including LASEK is also a valuable technique for patients with low myopia, a thinner cornea, or retinal pathology [10–12]. The main differences between the LASEK and LASIK procedures were the incidence rate of corneal haze and flap-related complications. The UDVA and CDVA could be reduced after LASIK due to flap-related complications including diffuse lamellar keratitis, epithelial ingrowth, flap traumatic shifting or avulsion [13]. Although several studies have reported corneal haze after surface ablation for the correction of high myopia [14, 15], there was no significant corneal haze between LASIK and LASEK for the treatment of low to moderate myopia [16, 17]. The MEL 90 has PRK and LASIK modes for laser ablation; the nomogram of PRK is different from the nomogram of LASIK, and PRK mode is more tissue saving than LASIK mode when treating the same refraction. Furthermore, there are the 250 Hz and 500 Hz pulse rates in LASIK mode, while PRK mode has only a 250 Hz pulse rate. In the current study, we have demonstrated it was feasible to perform LASEK procedures using the LASIK mode of the MEL 90 excimer laser with a 500 Hz pulse rate.

When compared to the MEL 80, the main differences of the MEL 90 were the pulse rate and ablation profile [9]. The treatment time of the 6 mm optical zone decreased from 3.2 s per diopter for the MEL 80 to 1.3 s per diopter for the MEL 90. A previous study found it to have a higher refractive predictability due to the faster ablation rate [9]. In the current study we further confirmed that predictability was higher after use of the MEL 90 when compared to the MEL 80, indicated by the higher *R* ^2^ value on the attempted versus achieved spherical equivalent scatter plot (Fig. 3a and b).

The MEL 90 offers a newly developed ablation profile called Triple-A, which consists of a basic profile in combination with a compensation algorithm for an enhanced energy correction. The enhanced energy correction in the Triple-A profile is stronger than that of ASA and TSA profiles. In two reports, this achieved better predictability compared with the ASA profile after LASIK and photorefractive keratectomy (PRK) [7, 8]. In our study we demonstrated better predictability achieved with the MEL 90 and Triple-A profile than with the MEL 80 and TSA profile after LASEK.

LASEK is the optimized mode for surface ablation, and several papers have reported good efficacy following LASEK for mild to moderate myopia [10, 18–20]. Since the MEL 90 was released, the safety and efficacy of the MEL 90 after LASIK has been reported by Reinstein et al. [9]. The safety, efficacy, and predictability of the MEL 90 after LASEK for mild to moderate myopia was worth investigation.

In our study all surgeries were uneventful and no definite intraoperative complications were observed. At postoperative 6 months, the safety index of the MEL 90 was 1.12 ± 0.15 and no eyes demonstrated a loss in CDVA, indicating the safety of LASEK with the MEL 90 Triple-A profile, which was consistent with the results after MEL 80 and previous studies [18, 19]. However, there were still some reports about postoperative complications such as haze, regression, and infection [21, 22]. Our research revealed that the epithelium survived and no patients complained of postoperative pain, which may be due to reduced dissociative time of the epithelial flap because of the faster pulse rate, and the activity of epithelial flap was retained to the greatest extent. This further increased safety and patient satisfaction following LASEK. The results in our study demonstrated the safety of using the Triple-A ablation profile with a 500 Hz pulse rate for a LASEK procedure.

The UDVA of all patients in the MEL 90 group at 1 week and 6 months was 20/25 or better and 20/20 or better, respectively, indicating the good efficacy of LASEK. The UDVA after LASEK was better than that after photorefractive keratectomy (PRK) in the early period after surgery [23, 24], which is characteristic of LASEK’s optimized surface ablation. Our results showed a faster recovery speed in UDVA when compared to the MEL 80 group (92% of eyes achieved 20/25 or better 1 week after surgery) and previous reports [20, 22, 25], which may be associated with the shorter ablation time, advanced ablation mode, and retained epithelial flap activity. PRK is no longer performed in our clinic in favor of LASEK, therefore the results following LASEK and PRK procedures were not compared in this study.

The SE diopter was 0.15 ± 0.41 D at 6 months after surgery with the MEL 90 excimer laser, which is similar to the results in the control group (0.19 ± 0.37 D, *P* = 0.613), and better than our previous results (−0.35 ± 0.41 D) [26], indicating the excellent predictability of the Triple-A ablation profile. Previous studies have reported regression and instability after LASEK [19, 21], while no such complications were observed in our study; this may be relative to the Triple-A ablation profile and a patient’s refraction. There are some limitations in this study. The refractive diopter was less than −6 D, and the follow-up period was relative short. Further studies with high myopia and a longer follow-up period will be conducted to demonstrate the outcomes after use of the MEL 90 excimer laser.

## Conclusions

In conclusion, the MEL 80 excimer laser and the MEL 90 excimer laser used with a 500 Hz pulse rate and the Triple-A profile showed comparable results in terms of efficacy, safety, and predictability for mild to moderate myopia in a LASEK procedure.

## Abbreviations

ASA: Aberration smart ablation
CDVA: Corrected distance visual acuity
HOAs: Higher-order aberrations
LASEK: Laser-assisted subepithelial keratectomy
LASIK: Laser-assisted in-situ keratomileusis
PRK: Photorefractive keratectomy
SE: Spherical equivalent
TSA: Tissue saving ablation
UDVA: Uncorrected distance visual acuity
